# 3D printed and spiral lithographically patterned erbium-doped polymer micro-waveguide amplifiers

**DOI:** 10.1038/s41598-021-00805-6

**Published:** 2021-10-28

**Authors:** Hongwei Gao, Huimin Li, G. F. R. Chen, P. Xing, M. C. Tan, D. T. H. Tan

**Affiliations:** 1 Photonics Devices and System Group, Singapore University of Technology and Design, 8 Somapah Rd, Singapore, 487372 Singapore; 2 Engineering Product Development Singapore University of Technology and Design, 8 Somapah Rd, Singapore, 487372 Singapore; 3 Institute of Microelectronics, A*STAR, 2 Fusionopolis Way, #08-02, Innovis Tower, Singapore, 138634 Singapore

**Keywords:** Optics and photonics, Integrated optics

## Abstract

Infrared (IR)-emitting RE doped materials have been extensively used to fabricate active components of integrated optical devices in various fields, such as fiber amplifiers, telecommunications, optoelectronics, and waveguides. Among various RE elements, trivalent erbium ions (Er 3+) are of great interest since their emissive behavior span the low loss telecommunication window of 1300–1650 nm. In this paper, we report two types of polymeric waveguide amplifiers. 8 cm long, lithographically patterned spiral waveguides provide 8 dB of gain using a 980 nm pump power of 95 mW. Gain is observed from 1530 to 1590 nm. We further report the first demonstration of polymeric waveguide amplifiers fabricated using 3D printing methods based on two-photon lithography, paving the way for rapid prototyping of active 3D printed devices and active photonic devices which may transcend planar limitations.

## Introduction

The advent of erbium doped fiber amplifiers (EDFAs) is one of the key reasons why telecommunications operating in the C-band have undergone global proliferation^1–3^. Owing to their possessing a gain spectrum in the C- and L-bands^4–6^, key technologies that drive telecommunications such as dense wavelength division multiplexing as standardized by the International Telecommunications Union (ITU) have become situated at these wavelengths. EDFAs allow propagation of light over long distances with limited attenuation. Typically, for these EDFAs, an optical pump located at 980 nm enables amplification of a signal within the 1530–1600 nm C-band region. Advancing from the widespread use of EDFAs in long-haul telecommunication networks, next generation on-chip waveguide amplifiers that offer higher gain in small device sizes and better integration with photonic devices have gained attention in recent years^4,7–22^. These chip-scale erbium-based amplifiers could be lithographically realized to provide amplification while still maintaining small form factors^8,18,23–25^.

Optical amplifiers relying on other gain mechanisms include semiconductor optical amplifiers (SOA), optical parametric amplifiers (OPA). SOAs rely on gain transitions from direct bandgap semiconductors to amplify light^26–28^. Compared to SOAs, Er-based amplifiers feature better optical amplification performance, generating less crosstalk and lower noise levels. OPAs on the other hand rely on parametric processes to transfer light from a pump to a signal, either through a χ^(2)^ or χ^(3)^ process^29–32^. Since the parametric amplification process requires phase matching for efficient amplification, Er-based amplifiers are arguably easier to implement, being agnostic to both the dispersion and detuning between pump and signal, as long as the signal is located within the Er-gain spectrum. In fiber-optic based communications, the Er-doped amplifier has proven to be the most widely deployed type of amplifier given the ease of deployment, high gain and relatively uniform gain spectrum. Therefore, their continued advancement in integrated optics may see a similar developmental trajectory formerly experienced by fiber-based Er-doped amplifiers.

In this manuscript, we demonstrate a lithographically defined, on-chip, erbium-doped polymer waveguide amplifier. The amplifier is formulated in a spiral with a total interaction length of 8 cm, and takes up a small footprint of 16 mm^2^. Using these waveguide amplifiers, we demonstrate a total gain of 8 dB and a length normalized gain of 1 dB/cm. Furthermore, we report the first demonstration of directly written, 3D printed polymer waveguide amplifiers, where the amplifier device is patterned and defined using two-photon polymerization lithography^33,34^. To the best of our knowledge, this is the first demonstration of both a spiral polymeric waveguide amplifier and a 3D printed polymeric amplifier. These results demonstrate both the promising amplification performance achieved by these polymeric waveguide amplifiers, and the flexibility of 3D printing in creating rapidly prototyped integrated active photonic components.

## Waveguide amplifier design and fabrication

**Figure 1:**
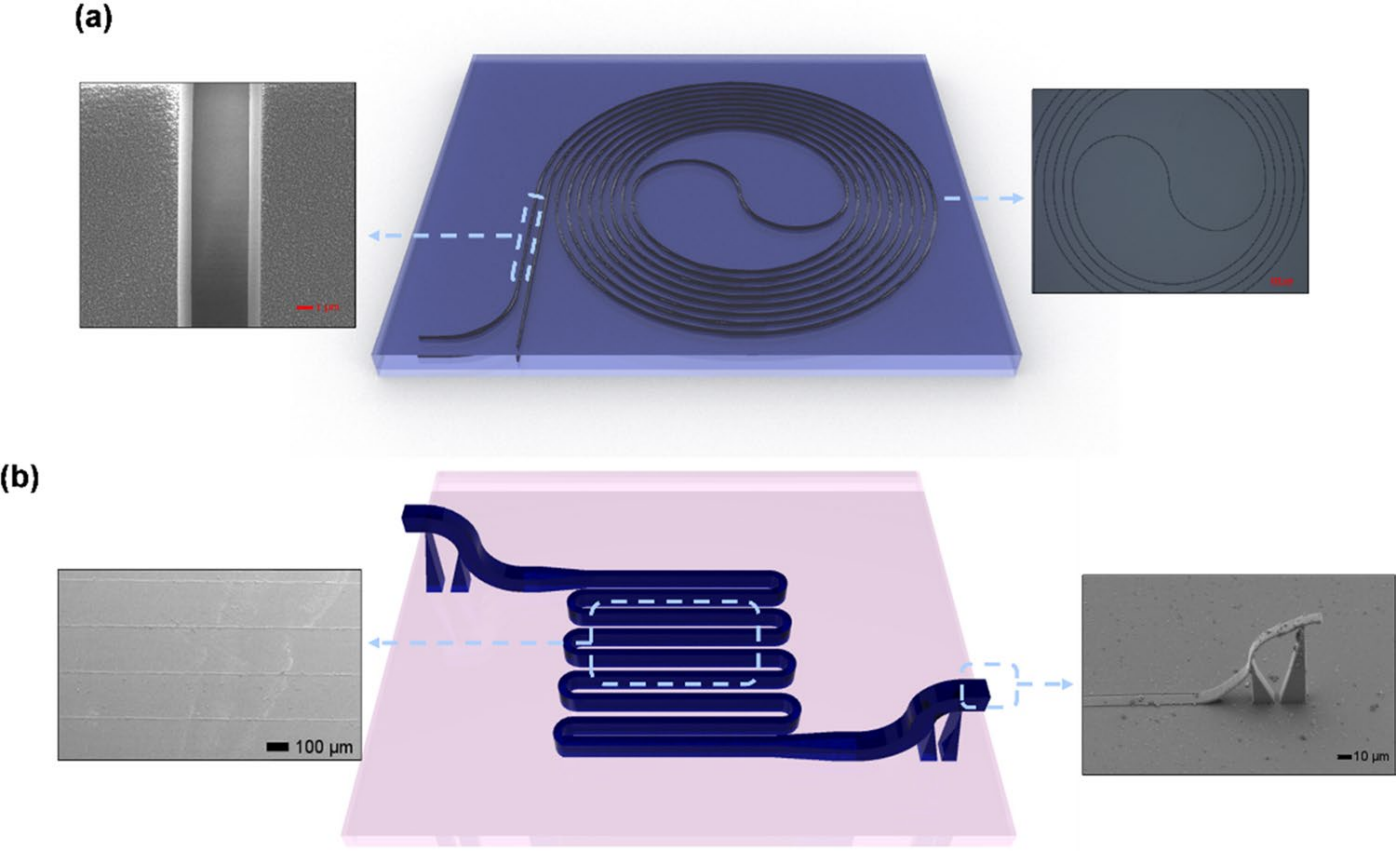
Device schematic of the polymer waveguide amplifier fabricated using (**a**) photolithography and (**b**) direct 3D printing. Inset of (**a**) shows a scanning electron micrograph (SEM) of the trenches and an optical micrograph of the fabricated trenches. Inset of (**b**) shows SEMs of the 3D printed waveguide and the 3D printed suspended couplers used for fiber-waveguide coupling.

The design of the waveguide amplifiers studied in this manuscript are shown in Fig. 1. The first class of devices consists of an SiO_2_ trench in the shape of a spiral waveguide (Fig. 1a). The trenches are 5 μm in width and 3 μm in depth. Erbium doped polymer is spin coated over the entire sample to fill the trenches and also to provide a cladding, 2 μm in height. In this first device, erbium-doped nanoparticles are dispersed in SU8, with a refractive index of 1.58. The modal profile of that results is therefore akin to an inverted ridge waveguide, where the core material making up the ridge is sandwiched between SiO_2_ (n = 1.46) on the bottom and air (n = 1) on the top. The second class of devices is shown in Fig. 1b. This class of 3D printed waveguide amplifiers consist of a waveguide with a width of 2 μm and a height of 2 μm. This structure is mainly air cladded on three sides, whereas the waveguide undercladding is SiO_2_. In this second device, erbium-doped nanoparticles with a lower concentration (10% that of the SU8 polymer) are dispersed in IP-Dip resist, with a refractive index of 1.53.

**Figure 2:**
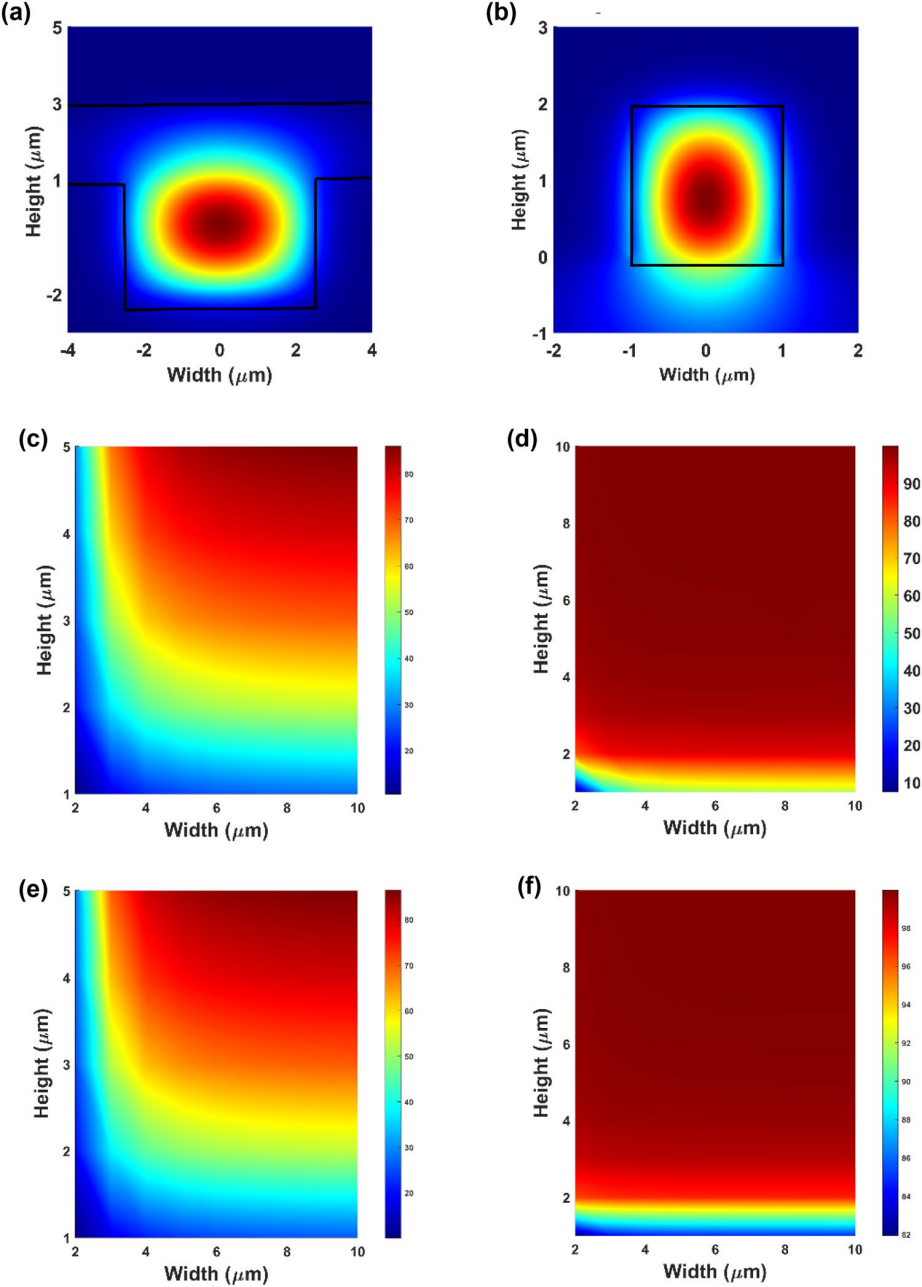
(**a**) The calculated TE mode profile for the spiral waveguide amplifier (cross section of W = 5 μm, H = 3 μm) and (**b**) the 3D polymer waveguide amplifier (cross section of W = 2 μm, H = 2 μm) at a wavelength of 1550 nm. (**c**) The calculated modal confinement (%) for the spiral waveguide amplifier and (**d**) 3D printed amplifier at 1550 nm. (**e**) The calculated modal confinement (%) for the spiral waveguide amplifier and (**f**) 3D printed amplifier at 980 nm.

**Figure 3:**
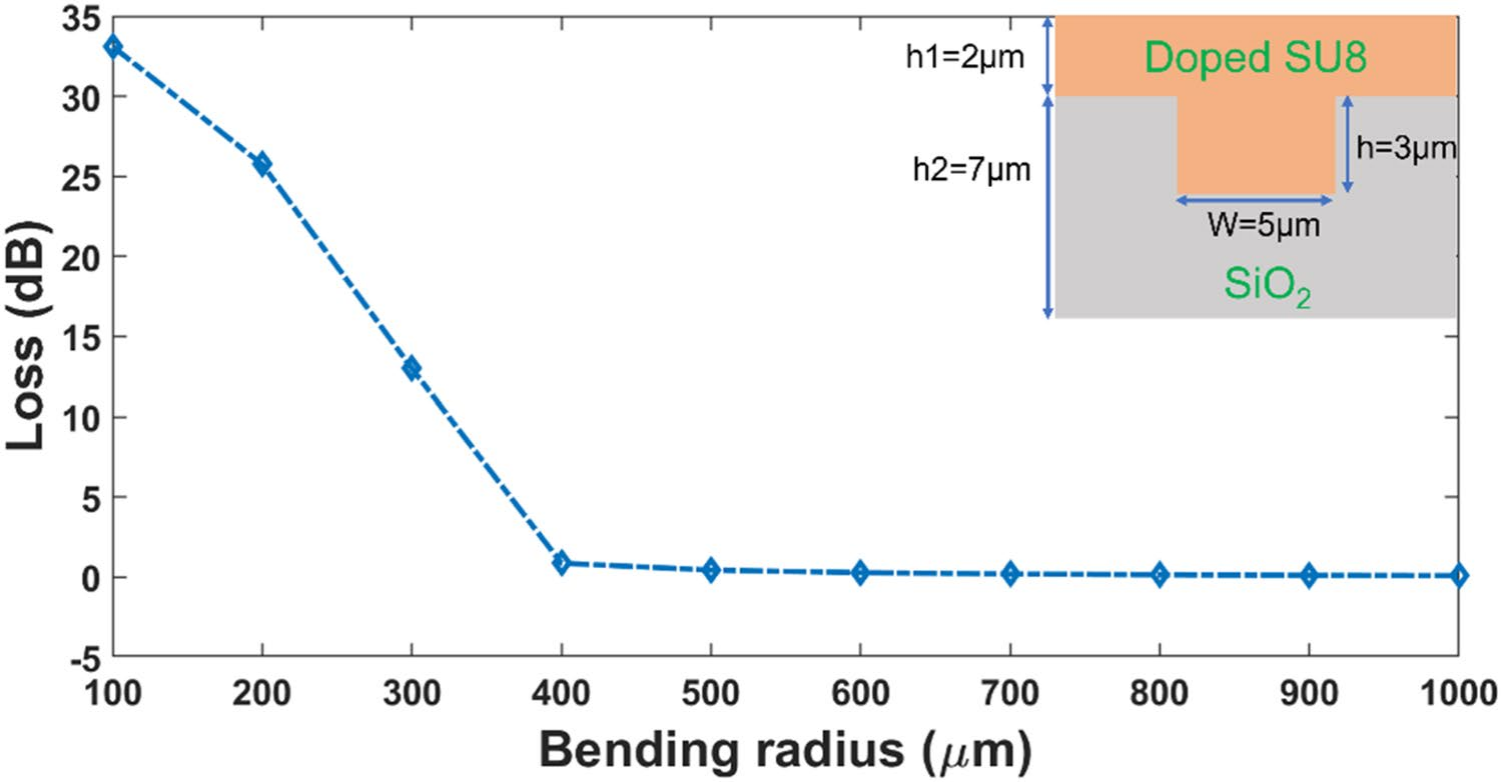
Calculated Bending Loss as a function of bending radius of photolithography fabricated polymer waveguide (inset shows a schematic of the structure).

We calculate the mode profiles and the percentage of light residing within the core material. The calculations are performed using a numerical solver based on the Finite Difference Eigenmode (FDE) method. The calculated modal profile of spiral waveguide and 3D printed waveguide at 1550 nm is shown in Fig. 2a,b. Optimal design requires that most of the light is confined in the core material, such that the interaction of the light with the Er-doped particles will be as large as possible. In both waveguides, it may be observed that most of the mode is well confined within the core. We further calculate the modal confinement factor for each of the waveguides as shown in Fig. 2c,d,e,f) for the spiral and 3D printed waveguides respectively. The modal confinement factor is calculated using the expression, $\frac{{\int }^{core}{|E(x,y)|}^{2}dxdy}{{\int }_{-\infty }^{\infty }{|E(x,y)|}^{2}dxdy}$, where *E*(*x*,*y*) is the electric field distribution of the eigenmode propagating in the waveguide amplifier. It is observed that for the 3D printed design used in our amplifiers, the confinement of the light at both 980 nm and 1550 nm within the core material where the Er-doped particles reside exceeds 95% (Fig. 2d,f). For the spiral waveguides, the height of the waveguides is constrained by the 7 μm SiO_2_ thickness we have in our wafers. The cladding material’s requirement is to (i) have a lower refractive index than the core and (ii) be sufficiently thick so as not to result in leakage of light into the cladding. From Fig. 2c,e, it is observed that a thicker trench depth leads to greater optical confinement. Therefore, we designed our waveguide to the largest possible trench depth (3 μm) while still ensuring no leakage of light into the cladding. For this design of width, W = 5 μm and height, H = 3 μm, most of the light is still confined within the Er-doped SU8 core: close to 70% for light at 980 nm and 1550 nm. The minimum bending radius of the spiral waveguide is also optimized using FDE solver calculations. In the simulation, a core size of 5 μm (W) by 3 μm (H), and an upper and under cladding with a thickness of 2 μm and 4 μm respectively are used. Doped SU8 is used for the upper cladding and SiO_2_ is used for the undercladding layer. The bending loss as a function of bending radius can be seen from Fig. 3. It is observed that the bending loss decreases as the bending radius is increased. When the bending radius increases up to 400 μm, the bending loss is close to 0 dB, indicating that the bending loss in our spiral waveguides which have bending radius exceeding 400 μm, is negligible.

In these two types of waveguide amplifiers, erbium-doped nanoparticles are dispersed in SU8 and IP-Dip respectively. The NaYF4:Yb,Er,Ce@NaYF4 core–shell RE nanoparticles (RENPs) are prepared by using a solvothermal decomposition method as reported elsewhere^7,35,36^. The RENPs original reaction mixture is taken and washed once with IPA, and then sonicated for 30 min and centrifuged at 6000 rpm for 10 min. Next, the RENPs are transferred and dispersed in DI water and sonicated for 10 min and centrifuged with 6000 rpm for 10 min. Subsequently, the RENPs are dispersed in DI water and dried using freeze dryer for 3 days. For SU8, the 5 mg freeze-dried RENPs are dispersed in 1 mL THF and then sonicated for 10 min. Next, the suspension of RENPs in THF are added to a solution containing 4 mg H-POSS-PMMA and 1 mL chloroform. After stirring for ~ 30 min, a solution of 25 µL SU8 in 1 mL chloroform is added. This solution is stirred for an additional for 30 min. The nanoparticle loading in SU8 is about 6 vol%. For the IP-Dip resist containing the erbium-doped nanoparticles, 15 mg freeze-dried RENPs are directly dispersed in 1 mL IP-Dip and stirred for 30 min, corresponding to the nanoparticle loading of around 0.6 vol%.

To fabricate the trenches, we start with a 7 μm SiO_2_ on Si substrate. Photoresist is first spin-coated on the sample before the spiral trenches are patterned using photolithography. Next, buffered oxide etchant (BOE) is used to isotropically etch the patterned spirals to define the trenches with a depth of 3 μm. Finally, the Er-doped nanoparticle SU8 polymer is spin-coated on the sample, thus creating the required index difference to guide light through the spirals. Importantly, our spiral design maintains a minimum bending radius of 500 μm in order to minimize scattering losses from the bends.

The second class of devices is fabricated using two-photon absorption lithography, in a direct 3D printed process. In this case, the RENP containing IP-Dip polymer (15 mg RENP in 1 mL IP-Dip resist) is dropped on an SiO_2_ substrate, after which it is patterned with femtosecond laser pulses generated from the commercial direct laser printing system (Nanoscribe Inc., Germany). The femtosecond laser is with wavelength of 780 nm, repetition rate of 80 MHz and pulse width of 100 fs. A × 63 NA1.4 oil immersion objective lens in Dip-in Laser Lithography (DiLL) configuration is used. The predesigned waveguide structure is prepared with Solidworks and Describe programming languages. After writing, the substrate is immersed in propylene glycol monomethyl ether acetate (PGMEA) for 10 min, isopropyl alchohol (IPA) for 5 min.

## Experimental characterization

**Figure 4:**
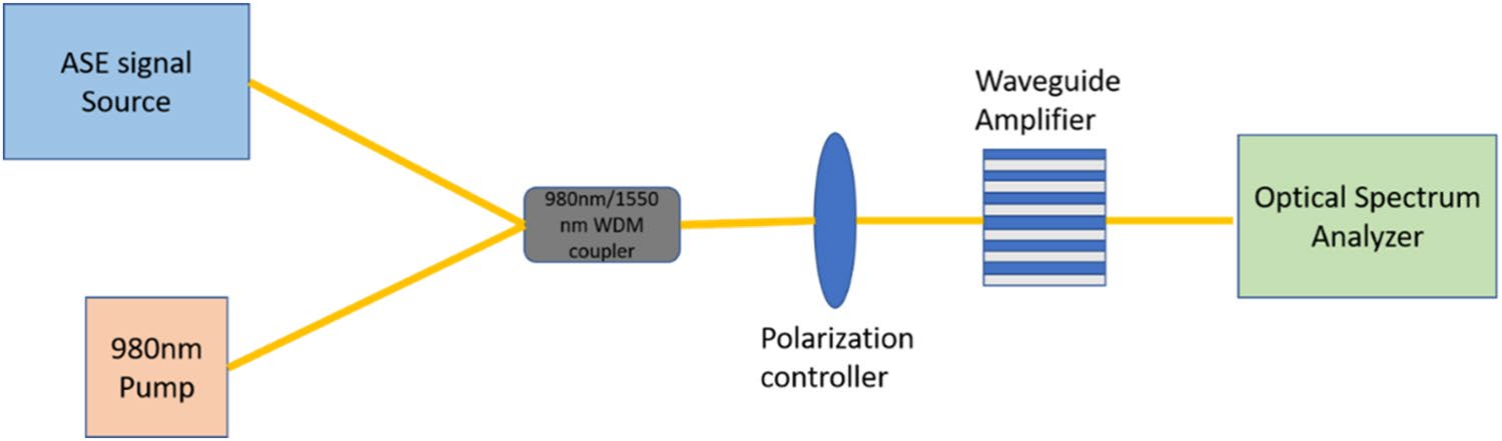
Waveguide device characterization setup used to measure the gain performance from our Er-doped waveguide amplifier.

We experimentally study the gain characteristics of the two different waveguide amplifiers. Figure 4 shows the schematic of the experimental setup. The experimental setup involves a 980 nm laser diode pump and a broadband source emitting between light in the C-band from 1530 to 1560 nm serving as the signal. Both light sources are combined using a wavelength division multiplexer before being coupled into the polymer waveguide amplifiers. The gain experienced by the signal is varied by controlling the amount of power emitted by the 980 nm laser diode pump. As it is known, when the pump power exceeds the threshold power, population inversion occurs between ^4^I_15/2_ and ^4^I_13/2_. Then, the Er3^+^ ions in the ^4^I_13/2_ level transition down to the ground level ^4^I_15/2_ and emit the photons with the same frequency as that of the signal, so the device realizes the amplification function for the signal.

In this report, the pump power was varied from 0 to 190 mW and the coupling loss at each fiber-waveguide interface was ~ 3 dB. It implies the actual coupled pump power into the waveguide was 0 to 95 mW. The input signal power was set to 0.5 mW through the amplified spontaneous emission (ASE) source. The relative gain was calculated using formula $G\left(dB\right)=10log\frac{{P}_{\mathrm{signal}(\mathrm{pump on})} }{{P}_{\mathrm{signal}(\mathrm{pump off})}}$, where *P*_signal(pump on)_ and *P*_signal(pump off)_ are the output signal powers as measured with and without pump power, respectively. The gain spectrum is measured using an optical spectrum analyzer (Yokogawa AQ6370D) with a wavelength resolution of 0.1 nm, which allows the gain to be quantified as a function of wavelength.

**Figure 5:**
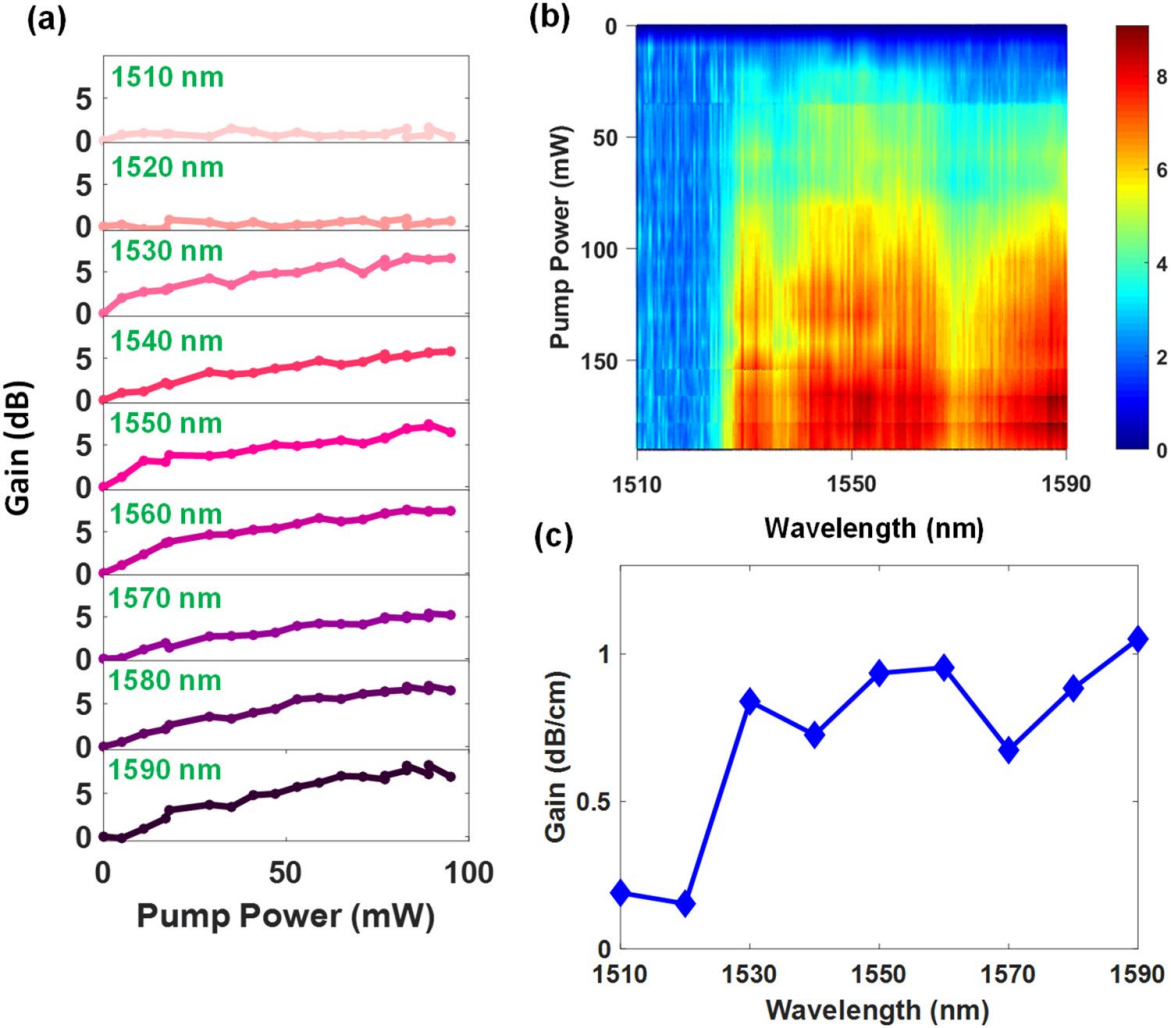
Gain properties for photolithography fabricated polymer waveguide amplifiers with a length of 8 cm (**a**) Experimentally measured gain as a function of pump power for wavelengths from 1510 to 1590 nm. (**b**) Gain spectrum as a function of pump power and wavelength from 1510 to 1590 nm. (**c**) Length normalized gain as a function of wavelength. The maximum gain achieved is 8.4 dB at a wavelength of 1590 nm.

Figure 5 shows the results of the optical characterization performed on the photolithography fabricated polymer waveguide amplifiers, where the gain process occurring in the Er-doped nanoparticles as a function of wavelength and pump power is shown in Fig. 5b. It is observed that the gain increases as the pump power increases for all wavelengths from 1530 to 1590 nm (Fig. 5a,b), in line with the larger number of photons available for down conversion from 980 nm to the 1550 nm region.

The maximum gain achieved at 1550 nm is 8 dB over an 8 cm device length, implying a length normalized gain of 1 dB/cm. Notably, good gain is also achieved at longer wavelengths up to 1590 nm, and a consequence of the efficiency of the down conversion: The gain efficiency at the longer wavelengths at 1580 nm and 1590 nm is comparable to that observed at 1550 nm (7.1 dB and 8.4 dB respectively at a pump power of 95 mW, as shown in Fig. 5c). This shows that the down conversion process is efficient over a wide bandwidth, providing efficient amplification from 1530 to 1590 nm. This result represents the first demonstration of an Er-doped spiral polymeric waveguide amplifier.

We note further that the polymer waveguide design minimizes scattering losses throughout the entire path length through the following ways (i) The minimum bending radius of 500 μm which renders bending losses to be negligible, and (ii) The modal engineering in the waveguide, which minimizes the amplitude of the electric-field in contact with the etched edges. (iii) Wet isotropic etching to define the spirals is a process that introduces less side-wall roughness compared to dry etching.

**Figure 6:**
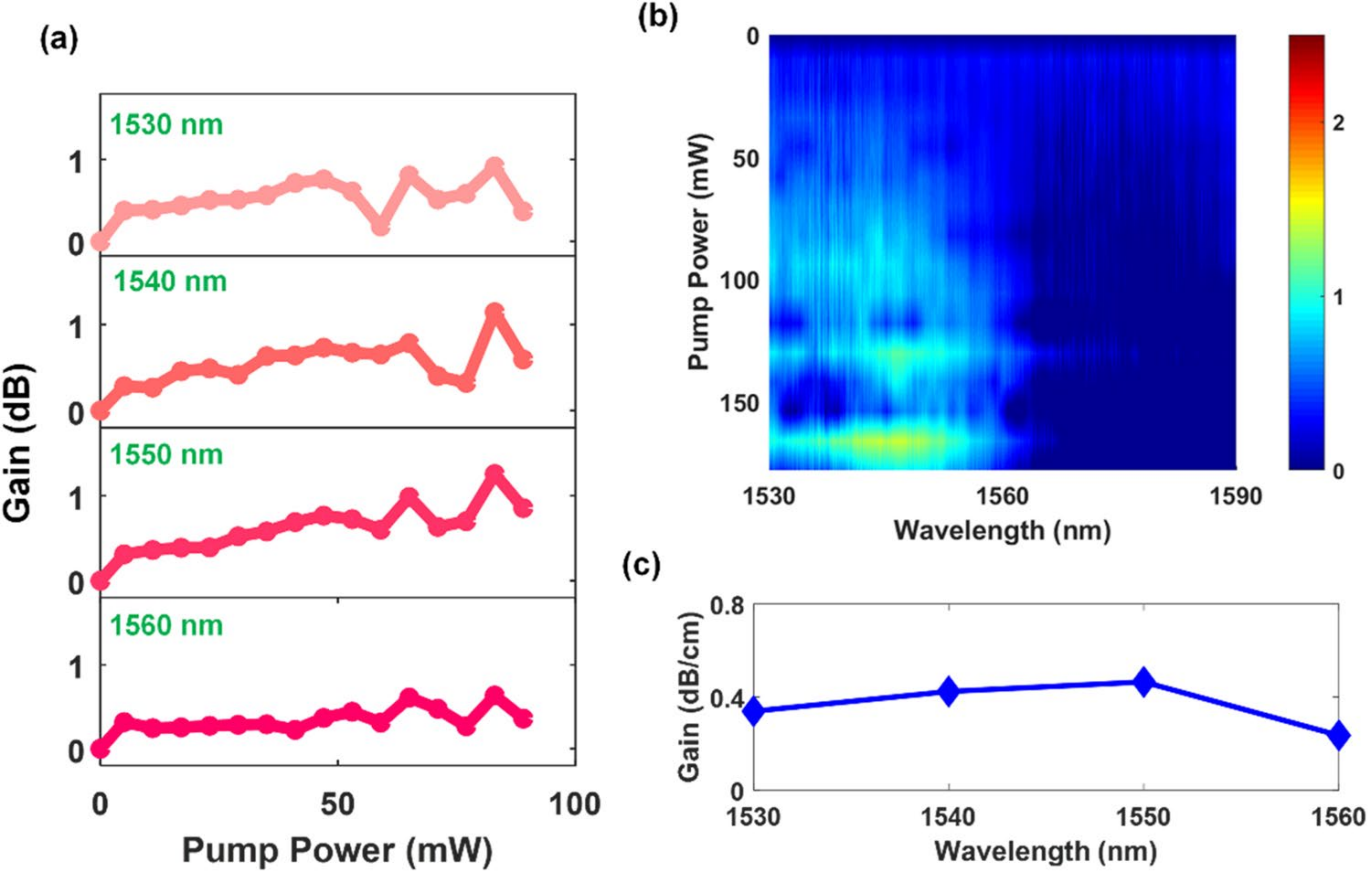
Gain properties for 3D printed amplifiers with a length of 2.7 cm. (**a**) Gain as a function of pump power for individual wavelengths. (**b**) Gain spectrum as a function of pump power and wavelength. (**c**) Length normalized gain as a function of wavelength. A maximum gain of 1.3 dB is achieved at a wavelength of 1550 nm.

Next, we experimentally characterize the second class of amplifiers: the 3D printed polymer waveguide amplifiers. These devices are fabricated in a single 3D printed step^33,34^. The integrated air-bridge waveguide couplers (shown in Fig. 1b) facilitate optical coupling with the input and output fibers, and have a coupling loss of ~ 2 dB for the material system used here. We note that in these waveguides, the concentration of the Er-doped nanoparticles is 10% that in the spiral amplifier. This lower concentration allows the patterning using 3D printing to be of higher quality, as a higher particle loading introduces more scattering sites which could cause the two-photon polymerization process to be affected. Therefore, we expect that the gain for these devices should be lower due to the lower particle loading. The 3D printed waveguide amplifier length is 2.7 cm and the gain performance is shown in Fig. 6.

A few points of interest may be highlighted. Firstly, the wavelength range over which gain is observed is 1530 to 1560 nm. Secondly, the maximum length normalized gain for the 3D printed waveguide amplifiers is 0.47 dB/cm at a wavelength of 1550 nm. The lower length normalized gain compared to the spiral waveguide amplifier is due to the lower concentration of optically active particles and the higher propagation loss of the waveguide fabricated by direct laser writing. Despite the particle concentration being tenfold lower than that used in the lithographically defined amplifiers, good amplification of 1.3 dB is achieved in the 3D printed polymer microwave guide amplifier. This result represents the first demonstration of a 3D printed, directly written erbium-doped waveguide amplifier.

## Discussion

In this manuscript, we report the first demonstration of 3D printed, erbium-doped polymer micro-waveguide amplifiers. The 3D printed amplifiers are fabricated in a single multi-photon lithographic step which allows design flexibility in accessing three-dimensional designs and the integration of air-bridge waveguide couplers. A maximum gain of 1.3 dB is achieved. Experimental characterization reveals that longer amplifier lengths allows gain to extend to the longer wavelengths, doubling the amplification bandwidth from 30 to 60 nm (1530–1590 nm). We further demonstrate lithographically defined spiral polymeric waveguide amplifiers with a maximum gain of 8.4 dB. The higher gain observed is attributed to a 10 × higher Er-nanoparticle loading in the polymer material. In addition, the efficient amplification observed may be attributed to the spiral waveguide design, which minimizes the amplitude of light interacting with the etched sidewalls and careful selection of the minimum bending radius in the spiral. Considerable progress has been made in Er-doped polymer-based waveguide amplifiers, with techniques such as trench dicing^8^ and photolithography^18,24^. In these prior demonstrations, the amplifiers were straight waveguides and did not incorporate a spiral configuration, thus limiting the footprint which is a typical performance metric for integrated photonics devices.

In addition, Er-doping has been successfully performed in a variety of other solid state waveguides^30–32^, including tellurite glasses^17,19,20^ and silicon nitride^21,22,37^. Relative pros and cons exist between polymer-based amplifiers and solid-state amplifiers. Polymer-based amplifiers benefit from the ease of low temperature, Er-doped nanoparticle synthesis with direct, single step device creation made possible now with 3D printed techniques as reported in this manuscript. Conversely, glass-based amplifiers have their own advantages from a manufacturing standpoint. Ref.^37^ for example reported the fabrication of silicon nitride based Er-doped amplifiers using a 300 mm silicon nitride CMOS process line. Summarily, the different approaches and platforms demonstrated showcase the longevity of Er-doped amplifiers which first existed in the fiber domain, and which have gradually made their way into integrated photonics. With the reaches of transceivers serving the data communications market becoming longer, and with the adoption of higher order modulation formats^38,39^, amplification to account for a limited link budget and ensure error free data could hasten the adoption of Er-doped amplifiers in commercial transceiver architectures.

The work reported here represents the first demonstration of both a spiral Er-doped polymer waveguide amplifier and a 3D-printed Er-doped waveguide amplifier. Importantly, the two approaches to fabricating Er-doped polymer micro-waveguide amplifiers showcase promising methods for realizing high gain on-chip waveguide amplifiers with flexible designs, extending typical planar structures to the third dimension.
